# Comparison of YouthCHAT, an Electronic Composite Psychosocial Screener, With a Clinician Interview Assessment for Young People: Randomized Controlled Trial

**DOI:** 10.2196/13911

**Published:** 2019-12-03

**Authors:** Hiran Thabrew, Simona D'Silva, Margot Darragh, Mary Goldfinch, Jake Meads, Felicity Goodyear-Smith

**Affiliations:** 1 Department of Psychological Medicine Faculty of Medical and Health Sciences University of Auckland Auckland New Zealand; 2 Department of General Practice Faculty of Medical and Health Sciences University of Auckland Auckland New Zealand; 3 Tamaki College Auckland New Zealand; 4 School of Health Science Faculty of Health and Environmental Sciences Auckland University of Technology Auckland New Zealand

**Keywords:** mass screening, adolescents, anxiety, depression, substance-related disorders, primary health care, school health services, eHealth

## Abstract

**Background:**

Psychosocial problems such as depression, anxiety, and substance abuse are common and burdensome in young people. In New Zealand, screening for such problems is undertaken routinely only with year 9 students in low-decile schools and opportunistically in pediatric settings using a nonvalidated and time-consuming clinician-administered Home, Education, Eating, Activities, Drugs and Alcohol, Sexuality, Suicide and Depression, Safety (HEEADSSS) interview. The Youth version, Case-finding and Help Assessment Tool (YouthCHAT) is a relatively new, locally developed, electronic tablet–based composite screener for identifying similar psychosocial issues to HEEADSSS

**Objective:**

This study aimed to compare the performance and acceptability of YouthCHAT with face-to-face HEEADSSS assessment among 13-year-old high school students.

**Methods:**

A counterbalanced randomized trial of YouthCHAT screening either before or after face-to-face HEEADSSS assessment was undertaken with 129 13-year-old New Zealand high school students of predominantly Māori and Pacific Island ethnicity. Main outcome measures were comparability of YouthCHAT and HEEADSSS completion times, detection rates, and acceptability to students and school nurses.

**Results:**

YouthCHAT screening was more than twice as fast as HEEADSSS assessment (mean 8.57 min vs mean 17.22 min; mean difference 8 min 25 seconds [range 6 min 20 seconds to 11 min 10 seconds]; *P*<.01) and detected more issues overall on comparable domains. For substance misuse and problems at home, both instruments were roughly comparable. YouthCHAT detected significantly more problems with eating or body image perception (70/110, 63.6% vs 25/110, 22.7%; *P*<.01), sexual health (24/110, 21.8% vs 10/110, 9.1%; *P*=.01), safety (65/110, 59.1% vs 17/110, 15.5%; *P*<.01), and physical inactivity (43/110, 39.1% vs 21/110, 19.1%; *P*<.01). HEEADSSS had a greater rate of detection for a broader set of mental health issues (30/110, 27%) than YouthCHAT (11/110, 10%; *P*=.001), which only assessed clinically relevant anxiety and depression. Assessment order made no significant difference to the duration of assessment or to the rates of YouthCHAT-detected positive screens for anxiety and depression. There were no significant differences in student acceptability survey results between the two assessments. Nurses identified that students found YouthCHAT easy to answer and that it helped students answer face-to-face questions, especially those of a sensitive nature. Difficulties encountered with YouthCHAT included occasional Wi-Fi connectivity and student literacy issues.

**Conclusions:**

This study provides preliminary evidence regarding the shorter administration time, detection rates, and acceptability of YouthCHAT as a school-based psychosocial screener for young people. Although further research is needed to confirm its effectiveness in other age and ethnic groups, YouthCHAT shows promise for aiding earlier identification and treatment of common psychosocial problems in young people, including possible use as part of an annual, school-based, holistic health check.

**Trial Registration:**

Australian New Zealand Clinical Trials Network Registry (ACTRN) ACTRN12616001243404p; https://www.anzctr.org.au/Trial/Registration/TrialReview.aspx?id=371422.

## Introduction

Psychosocial problems and risky health behaviors are significant issues for young people worldwide. In New Zealand, one-third of adolescents are affected by anxiety and depression [1]; the highest rate of suicide is among youth aged 15 to 24 years [2], and approximately one-fourth of high school students engage in hazardous alcohol use [3]. Mental health issues and risky health behaviors can lead to costly long-term health and social outcomes [4-6], and as such, local and World Health Organization policies emphasize the value of developing more effective tools and appropriately targeted and accessible services to identify and address the needs of young people [7-9]. At the same time, young people want a greater say in how services are designed and delivered and expect services to be diverse, contemporary, and responsive [10].

Home, Education, Eating, Activities, Drugs and Alcohol, Sexuality, Suicide and Depression, Safety (HEEADSSS) assessment is a clinician-administered interview-based assessment of young people that can identify mental health and substance use problems [11,12]. Currently, all year 9 (usually 13-year-old) students in low-decile schools (those with the highest proportion of students from low socioeconomic communities) and some attendees at primary care and pediatric services in New Zealand are screened for psychosocial problems via HEEADSSS assessment. Although HEEADSSS offers a straightforward, holistic, and gradual approach to assessing young people across many domains, it is a psychosocial interview rather than a screening tool. Drawbacks include its lack of validation for problem identification, the cost of resourcing, time required for administration (up to an hour per person), and variable quality depending on the skill and experience of the assessor.

**Figure 1:**
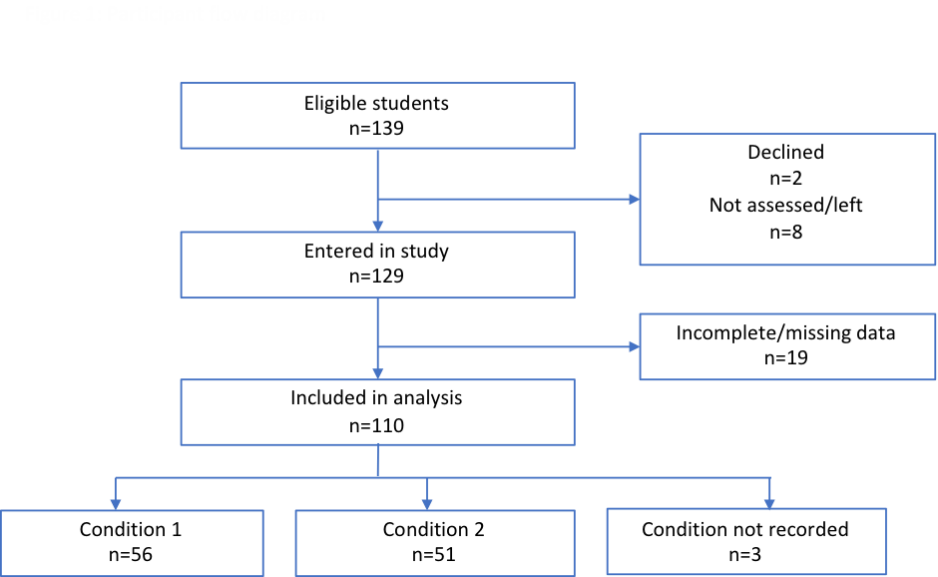
Participant flow diagram.

**Figure 2:**
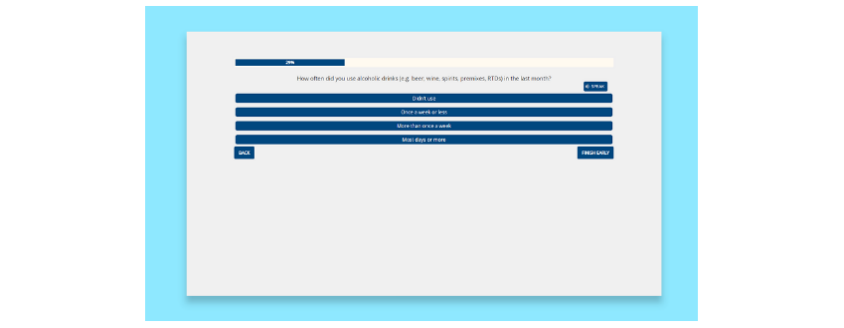
Youth Case-finding and Help Assessment Tool questions example.

The Youth version, Case-finding and Help Assessment Tool (YouthCHAT) [13,14] is a self-report, electronic screener that covers the following domains: smoking, drinking, recreational drug use (based on the Substances and Choices Scale, SACS) [15], problematic gambling, depression (based on the Patient Health Questionnaire-Adolescent Version, PHQ-A) [16,17], anxiety (based on the Generalized Anxiety Disorder-7 scale, GAD-7), sexual health, general stresses, exposure to abuse, behavior problems, anger management problems, eating problems, and physical activity [18]. For each positive domain screened, there is a *help* question that asks participants if they would like help either today or later. Responses to the *help* question support conversations between young people and their health providers about the issues they would like addressed, which facilitates shared decision-making, with increased likelihood that real sustained changes will be made (Figures 1 and 2).

Students complete YouthCHAT electronically on a device, and a summary report is immediately available for the nurse or other health provider through the electronic health record at the point of care. This includes positive or negative responses for each module, and where positive, the score and its interpretation when applicable (eg, depression: PHQ-A score 24=severe depression [20-27 out of a possible 27]) as well as whether help is wanted either now or later is included. A positive PQA-9 question triggers a red alert for self-harm. Where YouthCHAT indicates serious issues such as suicidal ideation, the nurse will intervene even when the students indicate they do not want help. Health providers using YouthCHAT are provided with stepped care resources for each module, tailored to their setting. Although detection of positive issues may increase referral rate and hence workload, this applies equally to HEEADSSS assessment.

YouthCHAT was developed via co-design with young people in primary care, youth, and school settings [13,14,19-22], and previous research has demonstrated its acceptability among young people of New Zealand European, Māori, and Pacific Island ethnicities [23] and identified that some students prefer disclosing sensitive information via electronic means rather than face-to-face means [14,24-27].

Electronic screening has been shown to provide consistent results and can lead to more disclosures and reduce staff time [28,29]. Arguments have been made for and against screening for mental health issues such as depression; however, screening has been found to be effective as long as it is linked to evidence-based interventions, not conducted as a *stand-alone* activity [30].

This study aimed to compare the performance and acceptability of YouthCHAT screening and HEEADSSS assessment for 13-year-old students attending a nurse-led clinic in a high school setting.

## Methods

### Trial Design

A randomized trial using a counterbalanced design was employed to deliver YouthCHAT screening either before or after face-to-face HEEADSSS assessment.

### Mapping of Youth Version, Case-Finding and Help Assessment Tool and Home, Education, Eating, Activities, Drugs and Alcohol, Sexuality, Suicide and Depression, Safety

Although YouthCHAT and HEEADSSS contained similar areas of assessment, it was necessary to map specific domains to each other for comparison of results. HEEADSSS includes domains on substance misuse, problem eating, sexual health, and physical activity, which are approximately equivalent to YouthCHAT modules. Questions regarding problems at home and safety were mapped for comparison (see Table 1). Although HEEADSSS *mental health* domain includes a number of nonspecific items such a sadness, grief, and difficulty sleeping, YouthCHAT mental health modules only comprised screening measures for depression and anxiety.

**Table 1:** Mapping Youth version, Case-finding and Help Assessment Tool (YouthCHAT) and Home, Education, Eating, Activities, Drugs and Alcohol, Sexuality, Suicide and Depression, Safety (HEEADSSS) assessment.

Item	YouthCHAT module	HEEADSSS domain
Substance misuse	Smoking or substance misuse (alcohol and drugs)—positive for A Stop *Smoking* In Schools Trial (ASSIST) or Substances and Choices (SACS) Scale	Positive responses to questions on alcohol and drugs
Problems with eating	Positive for problem eating module	Positive responses to questions on eating and weight
Mental health or distress	Depression or anxiety—positive for Patient Health Questionnaire-Adolescent Version (PHQ-A) or Generalized Anxiety Disorder-7 (GAD-7) scale	Positive responses to questions on low mood, self-harm, anxiety, suicidal thoughts, unresolved grief, sadness of historical event, and difficulty sleeping
Problems at home	Positive for “relationships with specific people in your life” or “issues at home, school or work”—from stress module	Positive responses to questions on problems at home
Sexual health	Positive response for sexual orientation, risky sexual behavior, and unwanted sex	Positive responses to questions on sexuality issues
Safety	Positive response to abuse or anger module, to questions on being bullied, or to violence in the stress module	Positive responses to questions on bullying, drunk driving, and other risky behaviors
Physical inactivity	Positive response to physical inactivity	Negative responses to questions on engaging in physical activity

For HEEADSSS assessment, there are no threshold scores—the assessor decides whether the response is positive for that domain or not. For YouthCHAT, responses are clearly positive or negative for each domain. Where there are added tools, cutoff points are as follows:

- ASSIST: 3 to 26 at risk of health and other problems from current pattern of smoking and greater than 26 at high risk of experiencing severe problems (health, social, financial, legal, and relationship) as a result of current pattern of smoking and likely to be dependent;
- SACS: 2 to 3 low-level problems with alcohol or drugs requiring further assessment and more than 3 problems with alcohol or drugs probably requiring treatment;
- PHQ-A: 10 to 14 moderate depression, 15 to 19 moderately severe depression, and 20 to 27 severe depression; and
- GAD-7: greater than 9 general anxiety disorder.

### Participants

All year 9 (13- to 14-year-old) students at a low-decile high school in Auckland, New Zealand, were invited to participate following the provision of written information about the study at the start of the school year and the completion of paired informed parental consent (using an opt-out process) and individual participant assent (as all students were aged <16 years). No students were excluded from the study. HEEADSSS assessment is mandatory for all year 9 students regardless of the study.

### Recruitment and Randomization

Participants were randomized to receive either HEEADSSS assessment by a school nurse followed by an electronic YouthCHAT screen (condition 1) or YouthCHAT followed by HEEADSSS assessment (condition 2). Clinic staff were provided with a random list from a computer-generated random numbers table, with consecutive sampling until all enrolled students had completed assessment. This took place during a planned break from class time when students receive their annually required HEEADSSS assessment. Review of results and any necessary follow-up was arranged by the school nurse immediately following the completion of YouthCHAT screening and HEEADSSS assessment.

### Outcomes

Primary outcome measures were (1) the time taken to complete YouthCHAT and HEEADSSS, (2) comparative detection rates for YouthCHAT and HEEADSSS for each issue, and (3) acceptability of YouthCHAT to students and staff. YouthCHAT data were collected electronically (completed on an electronic tablet by students), and encrypted results were securely stored on a central database. HEEADSSS results were entered into the electronic health record by school nurses. A subset of students completed paper-based acceptability questionnaires, and the 3 school nurses were interviewed individually.

### Analyses

Quantitative data were analyzed using Microsoft Excel 2013 and Statistical Package for the Social Sciences version 25 (SPSS v25). Analyses included basic descriptive statistics, between-intervention analyses undertaken with paired *t* tests (for numeric variables) or McNemar tests (for categorical variables), and between-condition nonparametric analyses undertaken with Mann-Whitney *U* tests. Distributions were checked for normality throughout.

Qualitative data were analyzed using a general inductive approach [31], with collated text independently coded by 2 researchers (HT and FG) to identify emerging themes. Discrepancies were resolved through an adjudication session.

Further methodological details are described in our trial protocol [32]. The study was approved by the New Zealand Northern Region Ethics Committee (16/CEN/137/AM03).

## Results

### Description of Participants

From the 139 eligible students, 129 assented. Electronic screening and face-to-face assessments were conducted between March and November 2017. There were incomplete or missing data for 19 students, giving a total sample size of 110 for analysis (81%; see Figure 1).

In addition, 63% (71/113) of the participants were of Pacific ethnicity, 29% (33/113) were of Māori ethnicity, and the remaining 8% (9/113) were of New Zealand European or other ethnicity. Moreover, 51% (58/113) of the participants were male, and the randomized condition numbers were similar, with 49% in condition 1 and 51% in condition 2. From the 32 students invited to participate in a focus group (8 during each term), 21 (66%) attended, with 3 groups of 5 participants and 1 group of 6 participants.

### Time Taken to Complete Youth Version, Case-Finding and Help Assessment Tool and Home, Education, Eating, Activities, Drugs and Alcohol, Sexuality, Suicide and Depression, Safety Assessment

HEEADSSS time data were missing for 19 students; therefore, the comparative time to complete analyses were conducted for 94 students. YouthCHAT took an average of 8 min 57 seconds (range 1 min 45 seconds to 54 min 15 seconds) to complete, compared with HEEADSSS with an average nearly double at 17 min 22 seconds (range 3 min to 45 min), giving a mean difference of 8 min 25 seconds (range 6 min 20 seconds to 11 min 10 seconds; *P*<.01). For several students, the Wi-Fi connection was lost for YouthCHAT, which may be reflected in the outlier durations of 25 min to 54 min, whereas the vast majority took 10 min or less.

### Detection Rates of Complete Youth Version, Case-Finding and Help Assessment Tool and Home, Education, Eating, Activities, Drugs and Alcohol, Sexuality, Suicide and Depression, Safety Assessment

The comparative detection rates are presented in Table 2. The 2 assessments had roughly similar detection rates for substance misuse and problems at home, but YouthCHAT detected significantly more issues around problems with eating or body image perception, safety, physical inactivity, and sexual health (all *P*<.01). HEEADSSS *mental health* category had a greater detection rate when compared with YouthCHAT-detected positive responses to the depression and anxiety tests (*P*<.01); however, no direct comparison between the assessments for only depression and anxiety rates was possible.

**Table 2:** Comparison between Youth version, Case-finding and Help Assessment Tool (YouthCHAT) screening and Home, Education, Eating, Activities, Drugs and Alcohol, Sexuality, Suicide and Depression, Safety (HEEADSSS) assessment.

Module/domain	YouthCHAT positive, n (%)	HEEADSSS positive, n (%)	*P* value^a^
Substance misuse	10 (9.1)	10 (9.1)	.99
Problems with eating or weight	70 (63.6)	25 (22.7)	<.01
Mental health/distress	11 (10.0)	30 (27.2)	.01
Problems at home	30 (27.3)	29 (26.3)	.72
Sexual health	24 (21.8)	10 (9.1)	.01
Safety	65 (59.1)	17 (15.4)	<.01
Physical inactivity	43 (39.1)	21 (19.1)	<.01

^a^*P* value from McNemar test.

### Effects of Randomization Order

The order in which students received YouthCHAT and HEEADSSS (condition 1 vs condition 2) made no significant difference to the duration of YouthCHAT assessment. For YouthCHAT, condition 1 took an average of 8 min 37 seconds (SD 6 min 54 seconds) and condition 2 took an average of 9 min 24 seconds (SD 8 min 26 seconds). For HEEADSSS, condition 1 took an average of 16 min 13 seconds (SD 8 min 42 seconds) and condition 2 took an average of 18 min 7 seconds (SD 9 min 49 seconds).

Similarly, the order made no significant difference to YouthCHAT-positive depression PHQ-A screen rates (both condition 1 and condition 2 had 5 positive screens; χ^2^_0.95_=0.0) or to anxiety GAD-7 rates (condition 1 had 3 screens and condition 2 had 2 screens; χ^2^_0.68_=0.25).

### Acceptability of Youth Version, Case-Finding and Help Assessment Tool and Home, Education, Eating, Activities, Drugs and Alcohol, Sexuality, Suicide and Depression, Safety Assessment

The results of the student acceptability survey completed by 21 students are shown in Table 3. There were no significant differences.

**Table 3:** Student acceptability of Youth version, Case-finding and Help Assessment Tool (YouthCHAT) screening and Home, Education, Eating, Activities, Drugs and Alcohol, Sexuality, Suicide and Depression, Safety (HEEADSSS) assessment (students attending focus groups, n=21).

Item		YouthCHAT, n (%)	HEEADSSS, n (%)	*P* value^a^
**Agreed to item**				
	Works for people my age	18 (85.7)	16 (76.1)	0.4
	I have time to think about my responses	16 (76.1)	11 (52.3)	>.99
	I felt safe answering the questions	14 (66.7)	12 (57.1)	0.5
	I talked about things that I wouldn’t have mentioned	11 (52.3)	9 (42.9)	0.7
	It’s easier to open up about my unhealthy behaviors and feelings	13 (61.9)	11 (52.3)	0.5
	It helped me identify the unhealthy behaviors and feelings I need help with	14 (66.7)	12 (57.1)	0.5
	Allowed my nurse to know about my unhealthy behaviors & feelings	13 (61.9)	14 (66.7)	0.7
	Has too many questions	6 (28.6)	6 (28.6)	0.99
	Questions are too personal	5 (23.8)	8 (38.1)	0.5
	I worried about the privacy of my information	9 (42.9)	6 (28.6)	0.3
	Takes too long	4 (19.0)	6 (28.6)	0.7
	Questions were difficult to understand	2 (9.5)	3 (14.2)	0.6
	Questions did not relate to me	1 (4.8)	2 (9.5)	0.6
	Is boring	2 (9.5)	3 (14.2)	0.6
	I felt embarrassed to talk to my nurse about my answers	6 (28.6)	7 (33.3)	0.7
	My nurse was judgmental about things I opened up about	1 (4.8)	2 (9.5)	0.6
**Objected to specific questions**				
	Substance misuse	9 (42.9)	4 (19.0)	0.2
	Problems with eating	0 (0.0)	2 (9.5)	0.99
	Problems at home	2 (9.5)	1 (4.8)	0.99
	Sexual health	8 (38.1)	3 (14.2)	0.8
	Safety	6 (28.6)	1 (4.8)	0.1
	Physical inactivity	2 (9.5)	1 (4.8)	0.99

^a^*P* value from chi-squared calculation with rates correction where n<10.

**Figure 3:**
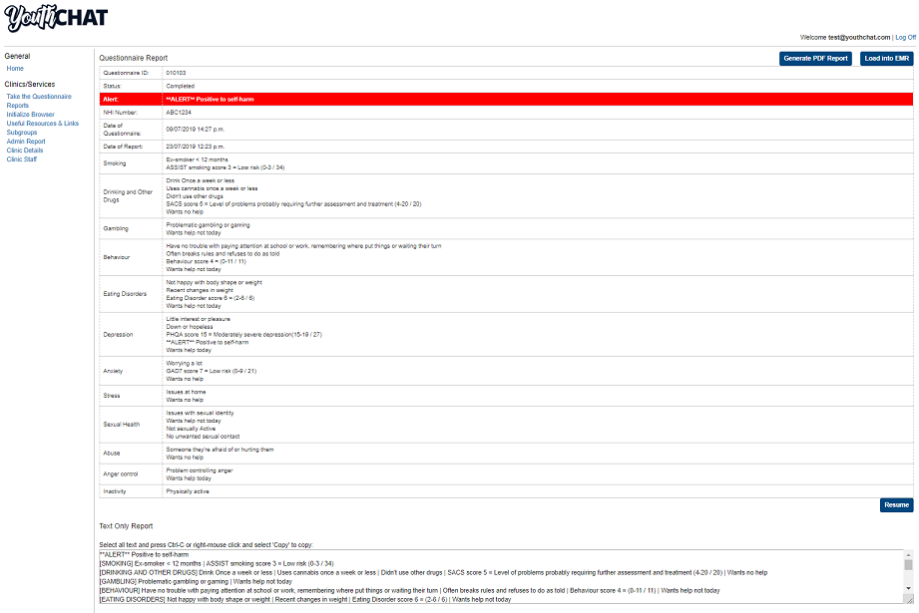
Youth Case-finding and Help Assessment Tool summary report.

A total of 4 key themes emerged from the analysis of the 3 nurse interviews (Table 4): (1) valuable tool, (2) difficulties with use, (3) comparing YouthCHAT with HEEADSSS, and (4) additional uses for YouthCHAT. In summary, students found YouthCHAT easy to understand, nurses liked its look and feel, it helped identify students at risk, and nurses found the summary report and the help question useful (Figures 2 and 3). Students did identify some difficulties with use, including Wi-Fi connectivity problems at times, and some students had literacy, language, or cognitive ability issues that were a barrier to its use. In comparison with HEEADSSS, nurses found YouthCHAT easier for students to answer, faster to administer, and helped students subsequently answer face-to-face questions. Nurses suggested additional uses, including repeating annually for a longitudinal picture and using opportunistically with at-risk students in all year groups.

**Table 4:** Nurses’ (N) views on Youth version, Case-finding and Help Assessment Tool.

Theme and subtheme		Example
**Valuable tool**		
	Questions easy to understand	“Most of the kids were able to answer the questions easily.” [N2]
	Look and feel	“The introductory video was awesome, was really nice and relaxed and helped the students engage.” [N3]
	Identifies students at risk	“Gathers information that you sometimes forget to ask.” [N1]
	Useful summary report	“I really liked the clinical summary at the end of YouthCHAT.1Other staff members (e.g., counsellors) thought it was excellent as well. A lot of information is extracted in 15 minutes, more than I could do 1:1.” [N2]
	Help question is useful	“I love the fact that it asks ‘Do you want help today…or in the future’ – we all know that nobody is going to change unless they want to, so it’s a good way of saving my time and theirs.” [N2]
**Difficulties with use**		
	Connectivity (Wi-Fi) issues	“We had some issues with connectivity.” [N1]
	Student literacy issues	“Literacy issues – those are the kinds of kids that give up early.” [N3]
	Hearing, language, cognitive ability, and other issues	“One guy with a hearing issue and the volume couldn’t go up high enough for him” [N3]; “Some of them had English skills that were not too good because they had just come over from the islands. Sometimes I get an interpreter in.” [N2]
**Comparing YouthCHAT** ^a^ **with** **Home, Education, Eating, Activities, Drugs and Alcohol, Sexuality, Suicide and Depression, Safety**		
	Easier to answer electronically	“Kids love [using e-tablet]. I think it is easier to say yes on an e-tablet (than face to face).” [N1]
	Faster to administer	“For me to do a HEEADSSS^b^, it takes so long and then to write it up, whereas YouthCHAT is so quick.” [N2]
	Helps with subsequent answering of face-to-face questions	“I think it’s better to give YouthCHAT first before talking with them face to face as it gets them in the groove, gives them time to get used to answering questions.” [N2]
**Additional uses for YouthCHAT**		
	Opportunistically with other school year groups	“Good to capture kids coming into the school halfway through the year. In fifteen minutes we can quickly capture where they are in their lives.” [N2]; “I would do it yearly.” [N1]
	Longitudinally, for example, repeat annually	“Be good to...follow up with them the next year.” [N1]
	Use by other staff	“I’d really like the rest of the school health team (counsellors, social workers, nurses, psychologist, addiction workers, GP) to be able to administer YouthCHAT, not just school nurses.” [N2]

^a^YouthCHAT: Youth version, Case-finding and Help Assessment Tool.

^b^HEEADSSS: Home, Education, Eating, Activtableities, Drugs and Alcohol, Sexuality, Suicide and Depression, Safety.

## Discussion

### Principal Findings and Comparison With Prior Work

Our results indicate that YouthCHAT is a timesaving, effective, and acceptable psychosocial screener for use in a high school setting. Even with the occasional Wi-Fi glitch, it was significantly faster to use.

In general, YouthCHAT had similar or significantly higher detection rates than HEEADSSS. Although the detection rate for mental health problems or distress was higher with HEEADSSS, this reflects poor mapping of the 2 assessments for this issue. YouthCHAT *mental health* consisted solely of positive scores for depression or anxiety on the PHQ-A or GAD-7, whereas the HEEADSSS domain also included many nonspecific issues such as low mood, distress, unresolved grief, sadness about a historical event, and difficulty sleeping; hence, it is to be expected that more students will score higher for *mental distress* than score positive for depression or anxiety with YouthCHAT. Rates of depression, anxiety, and substance use problems identified via YouthCHAT were in line with expectations for this age group [33,34].

Students revealed significantly more concerns via YouthCHAT than in their HEEADSSS assessment about sensitive issues such as their body weight and sexual health and safety issues such as bullying, violence, and anger. This is consistent with evidence that youth prefer to disclose sensitive information via electronic means, without fear of being judged [24-27]. Electronic screening helps them structure their thoughts and prioritize the issues for which they want help [24]. Despite the difference between groups not being statistically significant, students’ concerns regarding privacy of information and being asked questions about sensitive issues suggest that screening for psychosocial issues should always be undertaken in a careful manner and an appropriate setting.

To date, no other screening instrument has been shown to be effective for comprehensively identifying multiple psychosocial problems in young people. Reviews of individual instruments for identifying common psychological problems in young people have identified strengths and weaknesses of different psychometric tests and recommended that these instruments are reserved for targeted use within clinical settings [35,36]. Overall, 3 HEEADSSS-based electronic screeners, TickIT [37], myAssessment [38], and the Headspace Assessment Interview [39], have recently been demonstrated to be acceptable to users in hospital and youth clinic-based settings but have not been evaluated regarding their detection rates.

Given the temporally evolving and fluctuating nature of psychosocial issues during adolescence [40,41], routine (eg, annual) YouthCHAT screening is likely to increase the chance of early detection and intervention. Conducting YouthCHAT before a scheduled HEEADSSS assessment means that the latter needs to focus only on domains where YouthCHAT is positive. Such a *targeted* HEEADSSS approach will reduce the time taken and hence the cost of this assessment. Embedding YouthCHAT screening within a regular *holistic* school health check may also increase mental health literacy [42], normalize the management of psychosocial issues, and reduce stigma about help seeking [43]. Downstream benefits of early intervention may include improved social relationships, better engagement in education and employment, reduced involvement with the justice system, and lower rates of youth suicide [44].

### Strengths and Limitations

Strengths of this study include the comparison of YouthCHAT with an existing means of evaluating young people for psychosocial problems, the high response rate, and the collection of both student and staff perspectives on the use of electronic screening within a school environment. The restriction of participants to 13- to 14-year-olds and 3 school nurses from a single high school limits the generalizability of our findings. Owing to the variability in the time taken to complete both tests being considerably less than anticipated (ie, smaller SDs for the time taken to complete YouthCHAT screening and HEEADSSS assessment than expected), our power to detect a difference between the 2 interventions was higher than anticipated. Furthermore, there is a clear statistical difference between the interventions based on this sample; therefore, our final sample size was sufficient to answer our primary research questions. The inclusion of predominantly Māori and Pacific Island participants is both a strength and weakness of this study. Māori and Pacific people comprise 20% and 11%, respectively, of New Zealanders aged 10 to 17 years; hence, these ethnicities are oversampled. However, Māori and Pacific Island youth have higher rates of emotional difficulties [45,46], including depression [47] and suicide [48], yet they access specialist services at lower rates than other ethnicities [49], so early identification and intervention for these youth is key. Finally, the inability to directly map all the YouthCHAT modules to the HEEADSSS assessment domains limited the scope of comparison.

### Conclusions

YouthCHAT has been shown to be significantly quicker than HEEADSSS to administer, has a high detection rate of a range of psychosocial issues, and is acceptable to both students and staff. Its potential use is for both opportunistic and routine annual screening of high school students, especially those of low socioeconomic status. Next steps include its evaluation with students of different ages and in different types of school settings. Current evidence supports its use as a first-line screening instrument, which can be followed by a targeted HEEADSSS assessment where indicated.

## Appendix

Multimedia Appendix 1 CONSORT-EHEALTH checklist (V 1.6.1).

## Abbreviations

GAD-7: Generalized Anxiety Disorder-7
HEEADSSS: Home, Education, Eating, Activities, Drugs and Alcohol, Sexuality, Suicide and Depression, Safety
PHQ-A: Patient Health Questionnaire-Adolescent Version
SACS: Substances and Choices Scale
YouthCHAT: Youth version, Case-finding and Help Assessment Tool
