# Correction: Comparison of YouthCHAT, an Electronic Composite Psychosocial Screener, With a Clinician Interview Assessment for Young People: Randomized Trial

**DOI:** 10.2196/17339

**Published:** 2020-02-03

**Authors:** Hiran Thabrew, Simona D'Silva, Margot Darragh, Mary Goldfinch, Jake Meads, Felicity Goodyear-Smith

**Affiliations:** 1 Department of Psychological Medicine Faculty of Medical and Health Sciences University of Auckland Auckland New Zealand; 2 Department of General Practice Faculty of Medical and Health Sciences University of Auckland Auckland New Zealand; 3 Tamaki College Auckland New Zealand; 4 School of Health Science Faculty of Health and Environmental Sciences Auckland University of Technology Auckland New Zealand

In “Comparison of YouthCHAT, an Electronic Composite Psychosocial Screener, With a Clinician Interview Assessment for Young People: Randomized Trial” by Thabrew et al (J Med Internet Res 2019;21(12):e13911), there were two errors which were not identified during the proofing stage.

The original published title was incorrectly listed as:

Comparison of YouthCHAT, an Electronic Composite Psychosocial Screener, With a Clinician Interview Assessment for Young People: Randomized Controlled Trial

The correct title is:

Comparison of YouthCHAT, an Electronic Composite Psychosocial Screener, With a Clinician Interview Assessment for Young People: Randomized Trial

Although the study had two arms and randomization of participants did occur into two groups—one that completed intervention A before intervention B and the other that completed intervention B before intervention A—there was no control group.

Also, the academic degrees listed for Hiran Thabrew, Simona D’Silva, and Felicity Goodyear-Smith were provided incorrectly. On the original published manuscript their degrees were listed as “Hiran Thabrew, BSc, BM", “Simona D'Silva, BSc”, and "Felicity Goodyear-Smith, FRNZCGP, RCP, FFLM, MGP, MB CHB". The correct degree listings for these authors are as follows: “Hiran Thabrew, BSc, BM, FRACP, FRANZCP”, “Simona D'Silva, BHSc”, and "Felicity Goodyear-Smith, MBChB, MD, FRNZCGP(Dist)".

These corrections will appear in the online version of the paper on the JMIR website on February 3, 2020, together with the publication of this correction notice. Because this was made after submission to PubMed, PubMed Central, and other full-text repositories, the corrected article has also been resubmitted to those repositories.

