# Supplementary material for: Comparison of YouthCHAT, an Electronic Composite Psychosocial Screener, With a Clinician Interview Assessment for Young People: Randomized Controlled Trial
Source: J Med Internet Res. 2019 Dec 3;21(12):e13911. doi: 10.2196/13911 (PMC6918206; doi:10.2196/13911)
Supplement: Multimedia Appendix 1 [file jmir_v21i12e13911_app1.pdf]

CONSORT-EHEALTH Checklist V1.6.2 Report

Manuscript Number 13911

(based on CONSORT-EHEALTH V1.6), available at [http://tinyurl.com/consort-ehealth-v1-6].

Date completed

7/18/2019 21:27:46

by

Hiran Thabrew

Comparison of the Electronic Composite Psychosocial Screener YouthCHAT with a Clinician-Interview Assessment for Young People: A Randomised Trial

TITLE

1a-i) Identify the mode of delivery in the title

Comparison of the Electronic Composite Psychosocial Screener YouthCHAT with a Clinician-Interview Assessment for Young People: A Randomised Trial

1a-ii) Non-web-based components or important co-interventions in title

N/A

1a-iii) Primary condition or target group in the title

Young people mentioned in title

ABSTRACT

1b-i) Key features/functionalities/components of the intervention and comparator in the METHODS section of the ABSTRACT

A counter-balanced randomised trial of YouthCHAT screening either before or after face-to-face HEEADSSS assessment was undertaken with 129 New Zealand 13-year old high school students of predominantly Māori and Pacific Island ethnicity

1b-ii) Level of human involvement in the METHODS section of the ABSTRACT

N/A

1b-iii) Open vs. closed, web-based (self-assessment) vs. face-to-face assessments in the METHODS section of the ABSTRACT

Recruited from school "129 New Zealand 13-year old high school students of predominantly Māori and Pacific Island ethnicity"

1b-iv) RESULTS section in abstract must contain use data

All participants were received both interventions, so no subgroup numbers mentioned here. Key results described: YouthCHAT screening was more than twice as fast as HEEADSSS assessment (median 7.1 versus 17 minutes; t(92)=-7.197, P<.001).

1b-v) CONCLUSIONS/DISCUSSION in abstract for negative trials

N/A

INTRODUCTION

2a-i) Problem and the type of system/solution

Psychosocial problems and risky health behaviours are significant issues for young people worldwide. In New Zealand, a third of adolescents are affected by anxiety and depression[1]; the highest rate of suicide is among youth aged 15 to 24 years[2], and approximately a quarter of high school students engage in hazardous alcohol use [3]. Mental health issues and risky health behaviours can lead to costly long-term health and social outcomes [4-6],

2a-ii) Scientific background, rationale: What is known about the (type of) system

Electronic screening has been shown to provide consistent results, and can lead to more disclosures and reduce staff time [27, 28]. Arguments have been made for and against screening for mental health issues such as depression; however, screening has been found to be effective as long as it is linked to evidence-based interventions, not conducted as a 'stand-alone' activity [29].

Does your paper address CONSORT subitem 2b?

This study aimed to compare the performance and acceptability of YouthCHAT screening and HEEADSSS assessment for 13-year old students attending a nurse-led clinic in a high school setting.

METHODS

3a) CONSORT: Description of trial design (such as parallel, factorial) including allocation ratio

A randomised trial using a counterbalanced design was employed to deliver YouthCHAT screening either before or after face-to-face HEEADSSS assessment.

3b) CONSORT: Important changes to methods after trial commencement (such as eligibility criteria), with reasons

N/A

3b-i) Bug fixes, Downtimes, Content Changes

N/A

4a) CONSORT: Eligibility criteria for participants

All Year 9 (13-14 year old) students at a low decile high school in Auckland, New Zealand were invited to participate following the provision of written information about the study at the start of the school year and the completion of paired informed parental consent (using an opt-out process) and individual participant assent (as all students were under 16 years of age). No students were excluded from the study.

4a-i) Computer / Internet literacy

N/A

4a-ii) Open vs. closed, web-based vs. face-to-face assessments:

All Year 9 (13-14 year old) students at a low decile high school in Auckland, New Zealand were invited to participate following the provision of written information about the study at the start of the school year and the completion of paired informed parental consent (using an opt-out process) and individual participant assent (as all students were under 16 years of age).

4a-iii) Information giving during recruitment

All Year 9 (13-14 year old) students at a low decile high school in Auckland, New Zealand were invited to participate following the provision of written information about the study at the start of the school year and the completion of paired informed parental consent (using an opt-out process) and individual participant assent (as all students were under 16 years of age).

4b) CONSORT: Settings and locations where the data were collected

YouthCHAT data were collected electronically (completed on an iPad by students) and encrypted results were securely stored on a central database. HEEADSSS results were entered into the electronic health record by school nurses.

4b-i) Report if outcomes were (self-)assessed through online questionnaires

N/A

4b-ii) Report how institutional affiliations are displayed

N/A

5) CONSORT: Describe the interventions for each group with sufficient details to allow replication, including how and when they were actually administered

5-i) Mention names, credential, affiliations of the developers, sponsors, and owners

HT, SD, MD, MG and JM do not have any conflicts of interest to declare. FGS is the primary developer of YouthCHAT. HT, MD and FGS were supported by the University of Auckland for the submitted work.

5-ii) Describe the history/development process

Described in study protocol (ref 30)

5-iii) Revisions and updating

N/A

5-iv) Quality assurance methods

N/A

5-v) Ensure replicability by publishing the source code, and/or providing screenshots/screen-capture video, and/or providing flowcharts of the algorithms used

YouthCHAT modules described in Table 1

5-vi) Digital preservation

The Youth version of the electronic Case-Finding and Help Assessment Tool (YouthCHAT) [13, 14] is a self-report, electronic screener that covers the following domains: smoking, drinking, recreational drug use (based on the Substances and Choices Scale, SACS),[15] problematic gambling, depression (based on the Patient Health Questionnaire – Adolescent Version, PHQ-A) [16, 17], anxiety (based on the Generalized Anxiety Disorder scale GAD-7), sexual health, general stresses, exposure to abuse, behaviour problems, anger management problems, eating problems and physical activity (https://echat.org/projects/youthchat/Home).

5-vii) Access

All Year 9 (13-14 year old) students at a low decile high school in Auckland, New Zealand were invited to participate following the provision of written information about the study at the start of the school year and the completion of paired informed parental consent (using an opt-out process) and individual participant assent (as all students were under 16 years of age). No students were excluded from the study. Further details in study protocol (ref 30)

5-viii) Mode of delivery, features/functionalities/components of the intervention and comparator, and the theoretical framework

The Youth version of the electronic Case-Finding and Help Assessment Tool (YouthCHAT) [13, 14] is a self-report, electronic screener that covers the following domains: smoking, drinking, recreational drug use (based on the Substances and Choices Scale, SACS),[15] problematic gambling, depression (based on the Patient Health Questionnaire – Adolescent Version, PHQ-A) [16, 17], anxiety (based on the Generalized Anxiety Disorder scale GAD-7), sexual health, general stresses, exposure to abuse, behaviour problems, anger management problems, eating problems and physical activity (https://echat.org/projects/youthchat/Home). For each positive domain screened, there is a 'help' question that asks participants if they would like help either today or later. Responses to the "help" question support conversations between young people and their health providers about the issues they would like addressed, which facilitates shared decision-making, with increased likelihood that real sustained changes will be made.		
**5-ix) Describe use parameters**		
Only completed once per participant		
**5-x) Clarify the level of human involvement**		
Participants were randomised to receive either HEEADSSS assessment by a school nurse followed by YouthCHAT on an iPad (condition 1) or YouthCHAT followed by HEEADSSS assessment (condition 2) during a planned break from class time when students would usually have been receiving their HEEADSSS assessment. Review of results and any necessary follow-up was arranged by the school nurse immediately following the completion of YouthCHAT screening and HEEADSSS assessment.		
**5-xi) Report any prompts/reminders used**		
N/A , one off use supported by school nurse		
**5-xii) Describe any co-interventions (incl. training/support)**		
N/A		
**6a) CONSORT: Completely defined pre-specified primary and secondary outcome measures, including how and when they were assessed**		
Primary outcome measures were 1) the time taken to complete YouthCHAT and HEEADSSS, 2) comparative detection rates for YouthCHAT and HEEADSSS for each issue, and 3) acceptability of YouthCHAT to students and staff.		
**6a-i) Online questionnaires: describe if they were validated for online use and apply CHERRIES items to describe how the questionnaires were designed/deployed**		
No online questionnaires used		
**6a-ii) Describe whether and how "use" (including intensity of use/dosage) was defined/measured/monitored**		
Single use only		
**6a-iii) Describe whether, how, and when qualitative feedback from participants was obtained**		
A subset of students completed paper-based acceptability questionnaires and the three school nurses were interviewed individually.		
**6b) CONSORT: Any changes to trial outcomes after the trial commenced, with reasons**		
YouthCHAT data were collected electronically (completed on an iPad by students) and encrypted results were securely stored on a central database. HEEADSSS results were entered into the electronic health record by school nurses.		
**7a) CONSORT: How sample size was determined**		
**7a-i) Describe whether and how expected attrition was taken into account when calculating the sample size**		
Described in study protocol (ref 30)		
**7b) CONSORT: When applicable, explanation of any interim analyses and stopping guidelines**		
Primary outcome measures were 1) the time taken to complete YouthCHAT and HEEADSSS, 2) comparative detection rates for YouthCHAT and HEEADSSS for each issue, and 3) acceptability of YouthCHAT to students and staff.		
**8a) CONSORT: Method used to generate the random allocation sequence**		
Computer-generated randomisation described in study protocol (ref 30)		
**8b) CONSORT: Type of randomisation; details of any restriction (such as blocking and block size)**		
Computer-generated randomisation described in study protocol (ref 30)		
**9) CONSORT: Mechanism used to implement the random allocation sequence (such as sequentially numbered containers), describing any steps taken to conceal the sequence until interventions were assigned**		
Computer-generated randomisation described in study protocol (ref 30) - list provided to school nurses		
**10) CONSORT: Who generated the random allocation sequence, who enrolled participants, and who assigned participants to interventions**		
Computer-generated randomisation described in study protocol (ref 30) - list provided to school nurses		
**11a) CONSORT: Blinding - If done, who was blinded after assignment to interventions (for example, participants, care providers, those assessing outcomes) and how**		
**11a-i) Specify who was blinded, and who wasn't**		
N/A		
**11a-ii) Discuss e.g., whether participants knew which intervention was the "intervention of interest" and which one was the "comparator"**		
All participants completed both interventions, just in a different order		
**11b) CONSORT: If relevant, description of the similarity of interventions**		
Both interventions described in methods section		
**12a) CONSORT: Statistical methods used to compare groups for primary and secondary outcomes**		
Quantitative data was analysed using Microsoft Excel and SPSS (Statistical Software Package). Analyses included basic descriptive statistics, between-intervention analyses undertaken with paired t-tests (for numeric variables) or McNemar's tests (for categorical variables) and between-condition non-parametric analyses undertaken with Mann-Whitney U tests. Distributions were checked for normality throughout.		
**12a-i) Imputation techniques to deal with attrition / missing values**		
N/A		
**12b) CONSORT: Methods for additional analyses, such as subgroup analyses and adjusted analyses**		
N/A		
RESULTS		
**13a) CONSORT: For each group, the numbers of participants who were randomly assigned, received intended treatment, and were analysed for the primary outcome**		
From the 139 eligible students, 129 assented. Electronic screening and face to face assessments were carried out between March and November 2017. Incomplete or missing data for 16 students provided a total sample size of N =113 for analysis (81%) - see Figure 1. Demographics are shown in Table 2. Nearly two thirds were of Pacific and one third of Māori ethnicity, with roughly equal males and females. From the 32 students invited to participate in a focus group (eight each term), 21 (66%) attended, with three groups of five and one group of six.		
**13b) CONSORT: For each group, losses and exclusions after randomisation, together with reasons**		
From the 139 eligible students, 129 assented. Electronic screening and face to face assessments were carried out between March and November 2017. Incomplete or missing data for 16 students provided a total sample size of N =113 for analysis (81%) - see Figure 1. Demographics are shown in Table 2. Nearly two thirds were of Pacific and one third of Māori ethnicity, with roughly equal males and females. From the 32 students invited to participate in a focus group (eight each term), 21 (66%) attended, with three groups of five and one group of six.		
**13b-i) Attrition diagram**		
Single time point, so not relevant		
**14a) CONSORT: Dates defining the periods of recruitment and follow-up**		
Electronic screening and face to face assessments were carried out between March and November 2017.		
**14a-i) Indicate if critical "secular events" fell into the study period**		
N/A		
**14b) CONSORT: Why the trial ended or was stopped (early)**		
N/A		
**15) CONSORT: A table showing baseline demographic and clinical characteristics for each group**		
Demographics are shown in Table 2. Nearly two thirds were of Pacific and one third of Māori ethnicity, with roughly equal males and females. From the 32 students invited to participate in a focus group (eight each term), 21 (66%) attended, with three groups of five and one group of six.		
**15-i) Report demographics associated with digital divide issues**		
All participants of the same age		
**16a) CONSORT: For each group, number of participants (denominator) included in each analysis and whether the analysis was by original assigned groups**		
**16-i) Report multiple "denominators" and provide definitions**		
N/A		
**16-ii) Primary analysis should be intent-to-treat**		
N/A		
**17a) CONSORT: For each primary and secondary outcome, results for each group, and the estimated effect size and its precision (such as 95% confidence interval)**		
Paired samples t-tests found that YouthCHAT took significantly less time (505 seconds faster on average, 95% CI: 380 to 670, P<.001) to complete than HEEADSSS assessment.		
**17a-i) Presentation of process outcomes such as metrics of use and intensity of use**		
N/A		

**17b) CONSORT: For binary outcomes, presentation of both absolute and relative effect sizes is recommended**		
N/A		
**18) CONSORT: Results of any other analyses performed, including subgroup analyses and adjusted analyses, distinguishing pre-specified from exploratory**		
Qualitative analyses presented in tables 6,7		
**18-i) Subgroup analysis of comparing only users**		
N/A		
**19) CONSORT: All important harms or unintended effects in each group**		
For several students, the Wi-Fi connection was lost for YouthCHAT, which may be reflected in the outlier durations of 25 to 54 minutes, whereas the vast majority took 10 minutes or less.		
**19-i) Include privacy breaches, technical problems**		
N/A		
**19-ii) Include qualitative feedback from participants or observations from staff/researchers**		
Presented in tables 6,7		
DISCUSSION		
**20) CONSORT: Trial limitations, addressing sources of potential bias, imprecision, multiplicity of analyses**		
**20-i) Typical limitations in ehealth trials**		
Strengths of this study include the comparison of YouthCHAT with an existing means of evaluating young people for psychosocial problems; the high response rate and collection of both student and staff perspectives on the use of electronic screening within a school environment. The restriction of participants to 13-14 year olds and three school nurses from a single high school limit the generalisability of our findings. Due to the variability in the time taken to complete both tests being considerably less than anticipated (i.e. smaller standard deviations for the time taken to complete YouthCHAT screening and HEEADSSS assessment than expected), our power to detect a difference between the two interventions was higher than anticipated. Furthermore, there is a clear statistical difference between the interventions based on this sample. Therefore our final sample size was sufficient to answer our primary research questions. The inclusion of predominantly Māori and Pacific Island participants is both a strength and weakness of this study. Māori and Pacific peoples comprise 20% and 11% respectively of New Zealanders aged 10-17 years, hence these ethnicities are over-sampled. However, Māori and Pacific Island youth have higher rates of emotional difficulties [44] including depression [45] and suicide [46], yet access specialist services at lower rates than other ethnicities [47], so early identification and intervention for these youth is key. Finally, the inability to directly map the YouthCHAT modules to the HEEADSSS assessment domains limited the scope of comparison.		
**21) CONSORT: Generalisability (external validity, applicability) of the trial findings**		
**21-i) Generalizability to other populations**		
Strengths of this study include the comparison of YouthCHAT with an existing means of evaluating young people for psychosocial problems; the high response rate and collection of both student and staff perspectives on the use of electronic screening within a school environment. The restriction of participants to 13-14 year olds and three school nurses from a single high school limit the generalisability of our findings. Due to the variability in the time taken to complete both tests being considerably less than anticipated (i.e. smaller standard deviations for the time taken to complete YouthCHAT screening and HEEADSSS assessment than expected), our power to detect a difference between the two interventions was higher than anticipated. Furthermore, there is a clear statistical difference between the interventions based on this sample. Therefore our final sample size was sufficient to answer our primary research questions. The inclusion of predominantly Māori and Pacific Island participants is both a strength and weakness of this study. Māori and Pacific peoples comprise 20% and 11% respectively of New Zealanders aged 10-17 years, hence these ethnicities are over-sampled. However, Māori and Pacific Island youth have higher rates of emotional difficulties [44] including depression [45] and suicide [46], yet access specialist services at lower rates than other ethnicities [47], so early identification and intervention for these youth is key. Finally, the inability to directly map the YouthCHAT modules to the HEEADSSS assessment domains limited the scope of comparison.		
**21-ii) Discuss if there were elements in the RCT that would be different in a routine application setting**		
N/A		
**22) CONSORT: Interpretation consistent with results, balancing benefits and harms, and considering other relevant evidence**		
**22-i) Restate study questions and summarize the answers suggested by the data, starting with primary outcomes and process outcomes (use)**		
Our results indicate that YouthCHAT is a time-saving, effective and acceptable psychosocial screener for use in a high school setting, and in general had similar or significantly higher detection rates than HEEADSSS. While the detection rate for mental health problems or distress was higher with HEEADSSS, this reflects poor mapping of the two assessments for this issue. YouthCHAT 'mental health' consisted solely of positive scores for depression or anxiety on the PHQ-A or GAD-7, whereas the HEEADSSS domain also included many non-specific issues such as low mood, distress, unresolved grief, sadness about a historical event, and difficulty sleeping. Rates of depression, anxiety and substance use problems identified via YouthCHAT were in line with expectations for this age group [32, 33].		
**22-ii) Highlight unanswered new questions, suggest future research**		
Other information		
**23) CONSORT: Registration number and name of trial registry**		
This study was registered with the Australian New Zealand Clinical Trials Network Registry ACTRN12616001243404p.		
**24) CONSORT: Where the full trial protocol can be accessed, if available**		
Reference 30 (JMIR)		
**25) CONSORT: Sources of funding and other support (such as supply of drugs), role of funders**		
This study was generously funded by the Starship Foundation, New Zealand. The funder did not have any direct involvement in the design or conduct of the study, analysis of the data, or write-up of the results.		
**X26-i) Comment on ethics committee approval**		
The study was approved by the New Zealand Northern Region Ethics Committee (16/CEN/137/AM03).		
**x26-ii) Outline informed consent procedures**		
All Year 9 (13-14 year old) students at a low decile high school in Auckland, New Zealand were invited to participate following the provision of written information about the study at the start of the school year and the completion of paired informed parental consent (using an opt-out process) and individual participant assent (as all students were under 16 years of age).		
**X26-iii) Safety and security procedures**		
N/A		
**X27-i) State the relation of the study team towards the system being evaluated**		
HT, SD, MD, MG and JM do not have any conflicts of interest to declare. FGS is the primary developer of YouthCHAT. HT, MD and FGS were supported by the University of Auckland for the submitted work.		
